# Genome-Wide Identification of the Cation/Proton Antiporter (*CPA*) Gene Family and Expression Pattern Analysis Under Salt Stress in Winter Rapeseed (*Brassica rapa* L.)

**DOI:** 10.3390/ijms26073099

**Published:** 2025-03-27

**Authors:** Chunyang Han, Li Ma, Xiaolei Tao, Yintao Lian, Junyan Wu, Abbas Muhammad Fahim, Yanxia Xu, Xianliang Zhang, Lijun Liu, Gang Yang, Yuanyuan Pu, Tingting Fan, Wangtian Wang, Wancang Sun

**Affiliations:** 1 State Key Laboratory of Aridland Crop Science, Gansu Agricultural University, Lanzhou 730070, China; hannn216@163.com (C.H.); txl162185@163.com (X.T.); lianyintao@163.com (Y.L.); wujuny@gsau.edu.cn (J.W.); fahimabbaskhan@yahoo.com (A.M.F.); xyx7991@163.com (Y.X.); 18219919034@163.com (X.Z.); liulj198910@163.com (L.L.); yangang1018@163.com (G.Y.); vampirepyy@126.com (Y.P.); fantt@gsau.edu.cn (T.F.); wangw@gsau.edu.cn (W.W.); sunwanc@gsau.edu.cn (W.S.); 2 College of Agronomy, Gansu Agricultural University, Lanzhou 730070, China

**Keywords:** winter rapeseed (*Brassica rapa* L.), *CPA* gene family, expression analysis, Na^+^, K^+^ flow rate

## Abstract

The *CPA* gene family regulates ionic balance and pH homeostasis in cells, significantly contributing to plant stress tolerance. In this study, a total of 63 *BrCPA* gene family members were identified in the whole genome of *Brassica rapa* L. (*B. rapa*), and the three subfamily members were *BrNHX* (9), *BrKEA* (15), and *BrCHX* (39), respectively. The members of the *BrCPA* gene family encoded 303-1259 amino acids, with molecular weights in the range of 32,860.39~139,884.73 kDa, distributed on 10 chromosomes, and contained 17 conserved motifs, *BrNHX* and *BraKEA*, and the *BrCPA* gene family members had the same molecular weights on 10 chromosomes and contain 17 conserved motifs. The *BrNHX* and *BraKEA* subfamilies have more exons than the *BrCHX* subfamily. An analysis of promoter cis-acting elements in the *BrCPA* gene showed that members of this gene family contain TC-rich, LTR, MBS, and ARE stress response elements. In addition, transcriptome analysis revealed the expression of *CPA* genes in *B. rapa* under salt stress. The selected genes were verified by RT-qPCR. By detecting the Na^+^ and K^+^ flow rates in the root and chloroplast cells of salt-tolerant and salt-sensitive varieties after salt treatment, it was found that the rate of Na^+^ and K^+^ efflux from the root and chloroplast cells of salt-sensitive varieties was significantly higher than that of salt-tolerant varieties. This investigation marks the first systematic identification of the *CPA* gene family in *B. rapa*. This study further explores its expression patterns and the efflux rates of Na^+^ and K^+^ across salt-tolerant varieties, providing a theoretical basis for understanding the role of the *CPA* gene family in the salt stress response of *B. rapa*.

## 1. Introduction

Soil salinization represents one of the most critical challenges to agricultural productivity [1]. Salt ion concentrations in soil above normal levels can interfere with the normal functioning of plant cells, leading to abnormalities in basic metabolic processes such as seed germination and photosynthesis and serious damage to plant tissues, which in severe cases can lead to crop death and consequent crop yield loss [2,3]. In China, salinized land is mainly concentrated in arid and semi-arid areas as well as coastal areas [4,5,6]. *Brassica napus* and *B. rapa* are the main types of *B. rapa* cultivated in the northern region of China, while *B. rapa* is widely cultivated in the northwestern region of China because of its excellent cold and salt tolerance [7]. Because of its potential adaptive ability shown in saline and alkaline environments, in-depth investigation of the molecular mechanism of salt tolerance in *B. rapa* is the key to solving the current problem of the lack of *B. rapa* salt-tolerant varieties and also providing new ideas and methods for the efficient use of saline and alkaline land.

In order to resist the hazards caused by soil salinity, plants have evolved a variety of physiological mechanisms, such as ion uptake or efflux as well as ion homeostasis, in addition to morphological adaptations to maintain their normal growth [8]. The ability of plants to maintain ion homeostasis in the body under salt stress is crucial for their adaptation to high-salt environments, and many scholars have conducted studies on ion uptake under salt stress. For example, under high salt-stress conditions, plants can activate the Na^+^/H^+^ antiporter-mediated Na^+^ efflux mechanism on the plasma membrane to expel excess Na^+^ from the cytoplasm to the extracellular space, thereby maintaining intracellular ion homeostasis and enhancing their salt tolerance [9]. The overall superior salt tolerance of *Brassica napus* was achieved by matching higher osmotic tolerance with moderate tissue tolerance and superior K^+^ retention in the leaf pulp [10]. Siberian white spurge, on the other hand, resists ionic stress by increasing root K^+^ content and limiting Na^+^ efflux in the vesicles [11]. The above studies suggest that the ability of plants to maintain ion homeostasis after salt stress may be an important feature in measuring plant salt tolerance.

Plants have evolved the ability to regulate ion balance and pH homeostasis in the body throughout evolution, and these abilities are largely dependent on transmembrane ion channels and transporters on cells [12,13]. The *CPA* gene family maintains cytoplasmic ion homeostasis and enhances salt tolerance through Na^+^ efflux and compartmentalization [14]. The identification of *CPA* gene family members has been reported in a variety of plants, including *Arabidopsis thaliana* (*A. thaliana*) [15,16], potato [14], and soybean [17]. In plants, the *CPA* family of genes can be categorized into three subfamilies, namely the *NHX*, *KEA*, and *CHX* gene families [18]. Plants utilize Na^+^/H^+^ antiporters (Na^+^/H^+^ exchangers, NHX) to prevent the excessive accumulation of Na^+^ within their tissues [19]. In rice, salt stress upregulates the expression of *OsNHX1-5* and *OsNHX7*/*OsSOS1*, which can mitigate the sensitivity of rice to high Na^+^ and K^+^ concentrations [20,21]. In grapevines, *VvNHX1* also plays a crucial role in development and adaptation, influencing seed dormancy, growth, maturation, and stress responses [22]. KEA proteins in plants are K^+^ efflux reverse transporter proteins that are mainly responsible for K^+^ transport [23]. Aranda Sicilia et al. [24] demonstrated that *KEA1* and *KEA2* mediate K^+^ uptake/H^+^ loss to regulate stromal pH. CHX proteins play a crucial role in maintaining potassium and sodium ion homeostasis as well as regulating pH stability in plants [25]. The wild soybean *CHX19.3* gene enhances K^+^ uptake in overexpressing *A. thaliana* and positively regulates tolerance to high salinity and carbonate stress [26].

In this study, bioinformatics approaches were employed to comprehensively identify and analyze the *CPA* gene family in *B. rapa*, encompassing genome-wide identification, chromosomal localization, collinearity relationships, and expression patterns. Furthermore, real-time quantitative PCR (RT-qPCR) was utilized to validate the expression profiles of *CPA* family genes under salt-stress conditions. It provides a reference basis for further research on the mechanism of the response of *BrCPA* family genes to salt stress in *B. rapa*. The use of ion flow rate can be used as a physiological marker for evaluating the strength of salt tolerance in *B. rapa* varieties, providing technical support for the study of salt tolerance in *B. rapa* and the non-destructive identification of salt tolerance in important varietal resources, and thus accelerating the process of salt tolerance breeding in *B. rapa*.

## 2. Results

### 2.1. Analysis of Na^+^ and K^+^ Flow Rate Among Different Tissues Under Salt Treatment

**Figure 1:**
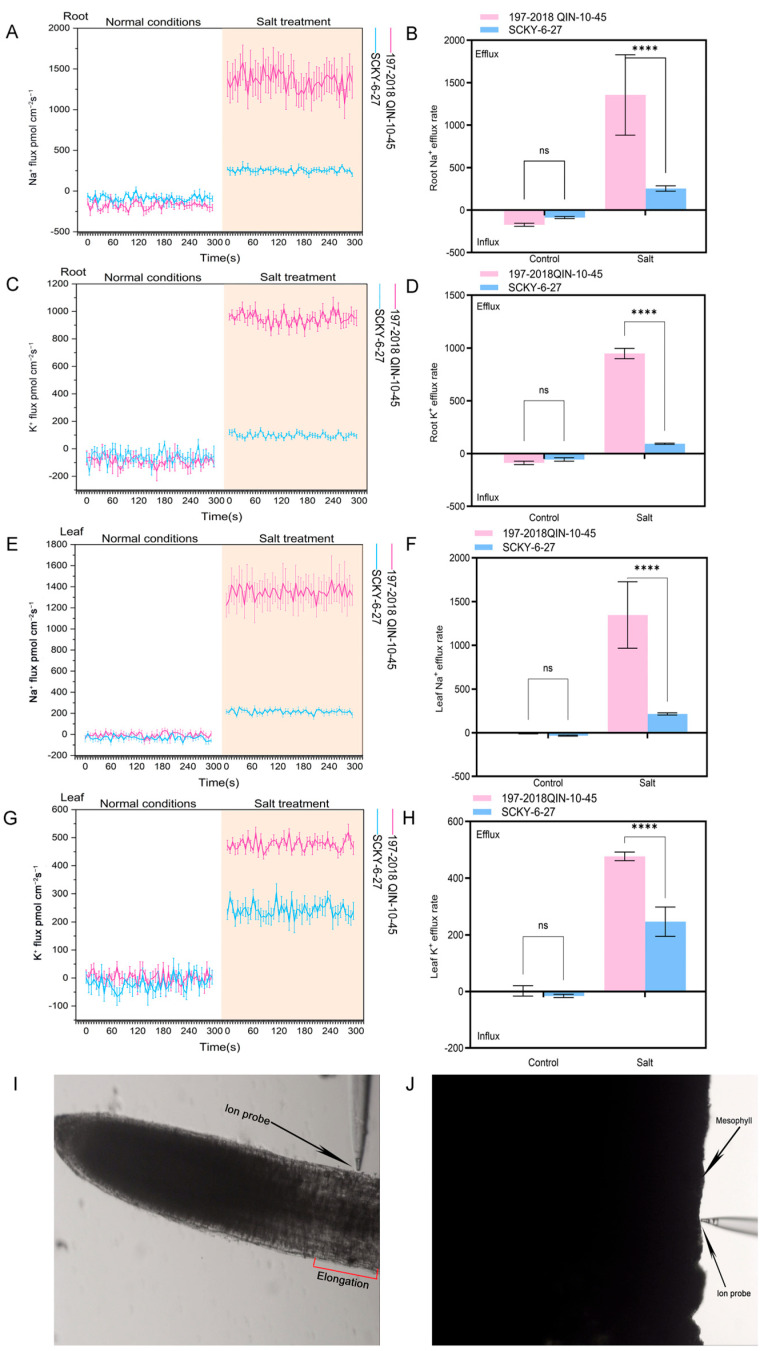
Non-invasive microtests were used to determine ion fluxes in roots and leaves under salt stress. (**A**,**B**) Net Na^+^ ion fluxes and mean Na^+^ ion fluxes in the extended region of *B. rapa* roots under control (no salt) versus salt-stress conditions; (**C**,**D**) Net K^+^ ion fluxes and mean K^+^ ion fluxes in the extended region of *B. rapa* roots under control versus salt-stress conditions; (**E**,**F**) Net Na^+^ ion fluxes and mean Na^+^ ion fluxes in the extended region of *B. rapa* leaves under control (no salt) versus salt-stress conditions; (**G**,**H**) Net K^+^ ion fluxes and mean K^+^ ion fluxes in the extended region of *B. rapa* leaves under control versus salt-stress conditions; (**I**) Representative measurement graph of Na^+^/K^+^ fluxes in the elongation zones of *B. rapa* roots; (**J**) Representative measurement graph of Na^+^/K^+^ fluxes of *B. rapa* leaves. Significant differences (****, *p* < 0.0001) were determined by unpaired two-tailed Student’s *t* tests between two groups using the SPSS 25.0 toolkit. ns, not significant.

In the present study, without salt treatment, the Na^+^ and K^+^ flow rates in the roots and leaf pulp cells of salt-tolerant *B. rapa* (SCKY-6-27) and salt-sensitive *B. rapa* (197-2018 Qin-10-45) were not significant. After 24 h of the 100 mM NaCl treatment, salt-sensitive *B. rapa* showed higher Na^+^, K^+^ flow rate in roots and chloroplasts compared with salt-tolerant *B. rapa* (Figure 1, Table S2). The instantaneous flow rates of Na^+^, K^+^ efflux in salt-sensitive *B. rapa* were higher than those in salt-tolerant *B. rapa* at all time points. The highest Na^+^ efflux rates were 1577.80 pmol cm^−2^s^−1^ for roots of salt-sensitive varieties and 313.15 pmol cm^−2^s^−1^ for roots of salt-tolerant varieties, respectively (Figure 1A,B). The highest K^+^ efflux rates were 1037.36 pmol cm^−2^s^−1^ for roots of salt-sensitive varieties and 139.23 pmol cm^−2^s^−1^ for roots of salt-tolerant varieties, respectively (Figure 1C,D). The highest Na^+^ efflux rate was 1436.12 pmol cm^−2^s^−1^ in the roots of salt-sensitive varieties and 246.50 pmol cm^−2^s^−1^ in the roots of salt-tolerant varieties (Figure 1E,F). The highest K^+^ efflux rate was 519.66 pmol cm^−2^s^−1^ in the roots of salt-sensitive varieties, 519.66 pmol cm^−2^s^−1^ in the roots of salt-tolerant varieties, and 246.50 pmol cm^−2^s^−1^ in the roots of salt-tolerant varieties (Figure 1G,H). The highest value of exocytosis rate of salt-tolerant variety roots was 304.61 pmol cm^−2^s^−1^. Comparing the exocytosis amplitude of the two varieties after salt treatment, the overall Na^+^ and K^+^ ions exocytosis amplitude of 197-2018 Qin-10-45 was significantly higher than that of SCKY-6-27. Suggesting that salt-tolerant materials exhibited reduced loss of Na^+^ and K^+^ under salt stress.

### 2.2. Identification of CPA Family Members in B. rapa

By comparing genomic databases of three generations of genes from *B. rapa*, a total of 63 *BrCPA* family members with complete conserved structural domains were screened (Table S3) and divided into three subfamilies: *BrNHX*, *BrKAE,* and *BrCHX*. These genes were named *BrNHX1-BrNHX9*, *BrKEA1-BrKEA15*, and *BrCHX1-BrCHX39* based on their homology to other species. These gene names, protein length (aa), molecular weight (MV), theoretical isoelectric point (pI), hydrophilicity index (GRAVY), and subcellular localization were determined. These genes vary in length and range in amino acid number from 303 (*BrKEA7*) to 1259 (*BrNHX5*). There are also differences in their molecular weights from 32,860.39 kDa (*BrKEA7*) to 139,884.73 kDa (*BrNHX5*), and the estimated isoelectric points were found to range from 5.14 (*BrKEA8*) to 9.23 (*BrCHX3*), of which, 37 proteins were acidic, and 26 were basic. Among the BrCPA proteins, a vast majority of proteins belonged to hydrophobic proteins, and only *BrKEA11, BrKEA12*, *BrKEA13*, and *BrKEA14* belonged to hydrophilic proteins, ranging from −0.018 (*BrKEA12*) to 0.677 (*BrKEA5/BrKEA6*). Subcellular localization predictions tell us that most genes are present in the cytoplasm, consistent with functioning as transporters to maintain ion homeostasis; in addition, a few of the gene members were located in the nucleus and chloroplasts.

### 2.3. Phylogenetic Tree and Chromosome Localization Analysis

**Figure 2:**
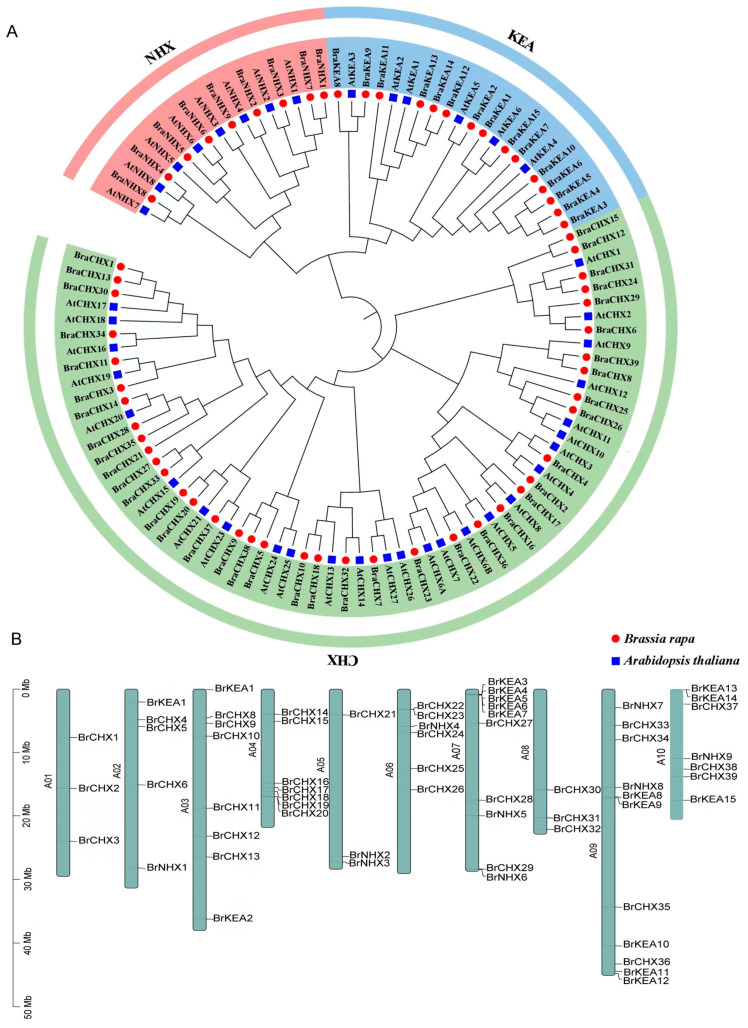
(**A**). Phylogenetic tree of *CPA* family members in *A. thaliana* and *B. rapa* red circles represent *B. rapa*, blue squares represent *A. thaliana*. (**B**). Chromosomal localization of *BrCPA* gene family members.

To investigate the evolutionary links among all *BrCPA* members, we retrieved the complete sequences of CPA proteins from the genomes of *B. rapa* and *A. thaliana* and compared them to construct a phylogenetic tree of *CPA* members. A total of 105 CPA proteins were screened from *B. rapa* and *A. thaliana*, and all *CPA* members were categorized into three subfamilies, *NHX*, *KEA*, and *CHX*, with *BrCPA* members distributed in each subfamily (Figure 2A). The *CHX* subfamily has the highest number of gene members, and the *NHX* subfamily has the lowest number of gene members. The phylogenetic tree shows that the *CPA* gene family exhibits a very high degree of conservation across species. The *BrCPA* family members were unevenly distributed on all chromosomes of *B. rapa* (Figure 2B). Among them, chromosome 9 (A09) had the highest number of genes, with 11 genes, respectively. In contrast, the distributions of genes on chromosome 1 (A01), chromosome 5 (A05), and chromosome 8 (A08) were the lowest, with only three genes. In addition, we found five tandem duplicate gene pairs in the *BrCPA* genes, suggesting a similar function.

### 2.4. Characterization of Cis-Acting Elements in the BrCPA Gene Promoter

**Figure 3:**
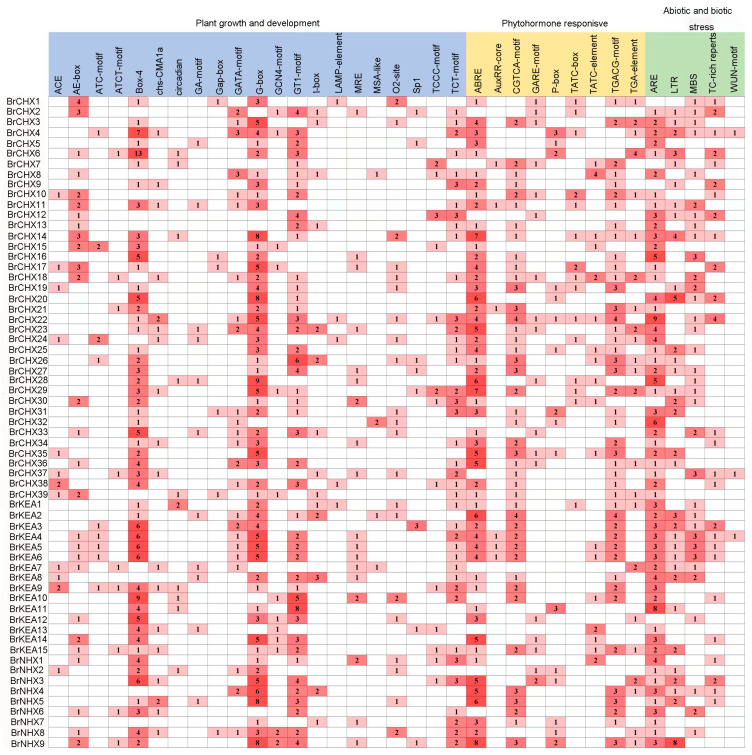
The CPA gene family promoter cis-acting elements. The color intensity and numerical values in the figure represent the quantity of cis-acting elements corresponding to each gene.

Promoter *cis*-acting elements are crucial binding sites for transcription initiation proteins and significantly influence gene expression regulation. To investigate the types of *cis*-acting regulatory elements in the *BrCPA* promoter, we searched and analyzed 2000 bp sequences upstream of the initiation codon (ATG) of all *BrCPAs*. A total of 35 common elements were identified in the promoter region of *BrCPA* to investigate their potential biological functions. From Figure 3, it is clear that most *CPA* gene family members are co-regulated by a variety of elements. They are closely linked to plant growth and development processes and also play a role in plant response to hormone regulation as well as in coping with abiotic stresses. In addition to containing a large number of basic promoter elements, there are light-responsive elements (ACE, G-box, and GT1-motif), stress-responsive elements (TC-rich, LTR, MBS, and ARE), and hormone-responsive elements (ABRE, TCA-element, TATC-box, and P-box). These results suggest that *CPA* genes are not only regulated by light signaling and phytohormones but may also participate in the signaling pathway of plant response to stress through these action elements and play a role in plant responses to stress.

### 2.5. Motif Composition and Gene Structure of the BrCPA Gene Family

**Figure 4:**
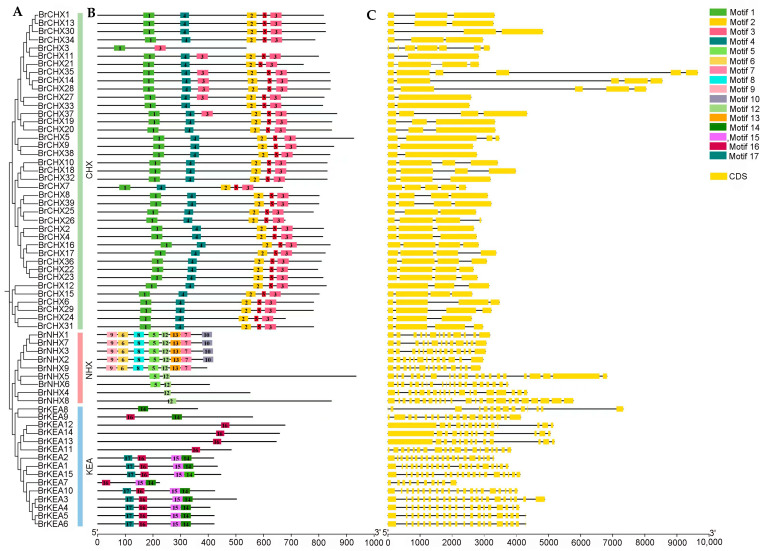
Gene structure analysis. (**A**). Phylogenetic tree of *CPA* family members in *B. rapa*. (**B**). Conserved motif distribution of BrCPA. (**C**). Gene structure map of BrCPA.

Based on the pfam structural domains of the three subfamilies of the *CPA* gene family, we categorized the 63 identified BrCPA proteins into three subfamilies (Figure 4). Each gene has between one and eight motifs, and motif5 is distributed in the *CHX* and *NHX* families, suggesting an evolutionary correlation between these two subfamilies. While most motifs were distributed in only one family, such as motif1, motif2, motif3, and motif4 in the *CHX* family, motif6, motif7, motif8, motif9, motif10, motif12, and motif13 in the *NHX* family, and motif14, motif15, motif16, and motif17 are distributed in the *KEA* family. Most *BrCPA* members have similar motif composition in the same subfamily, suggesting that these proteins are relatively conserved among themselves. In addition, the number of motifs exhibited variation among different subfamilies suggests that distinct subfamilies may exhibit diverse functional roles. The gene structure of *BrCPAs* was then analyzed, and the results showed that the sequence lengths, as well as the number of introns and exons, were significantly different among the *BrCPA* members, with shorter exons in the *CHX* family members and longer exons in the *NHX* and *KEA* family members, and all of the members were free of UTR regions.

### 2.6. Analysis of Intra and Inter-Species Covariance in BrCPA Genes

**Figure 5:**
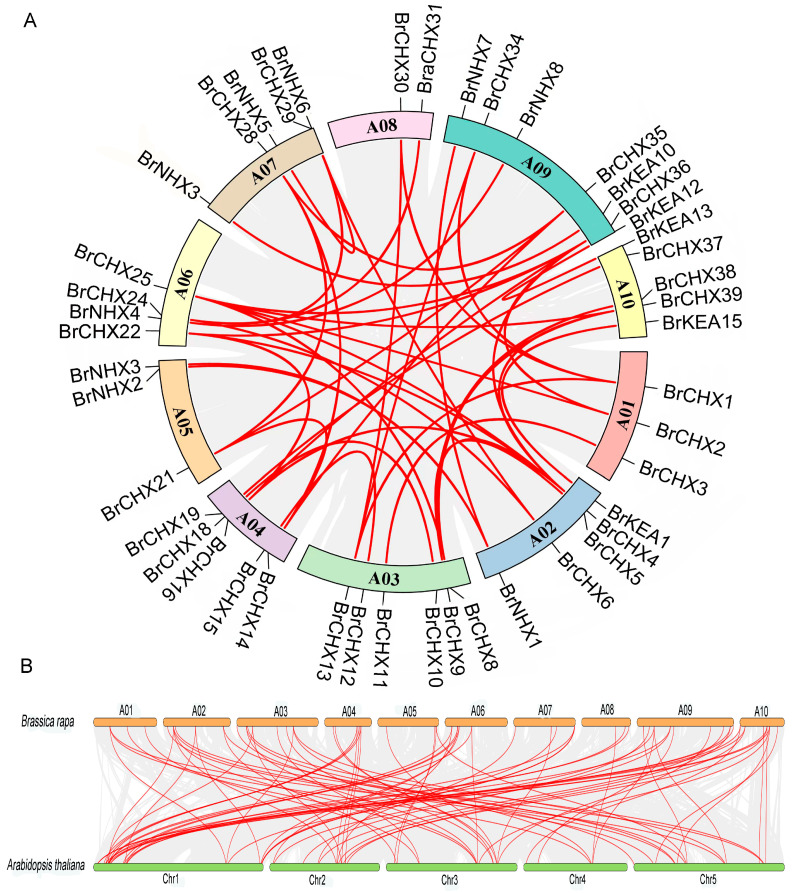
Replication relationships among BrCPA gene family fragments. (**A**). Analysis of BrCPA gene intraspecific replication events. (**B**). BrCPA genes and *A. thaliana* genomic duplication events.

By covariance analysis of the *BrCPA* gene within the *B. rapa* species (Figure 5A), a total of 25 pairs of co-linear *BrCPA* genes were identified. Co-linearity of *BrCPA* genes was observed on all chromosomes, with tandem duplicates accounting for 39.7% of the gene family. Furthermore, the *CPA* genes of *B. rapa* and Arabidopsis were compared and analyzed by the synteny block method to explore the evolutionary mechanism of *BrCPA* members. (Figure 5B). Upon exploring the evolutionary relationship between BrCPA proteins and different species, the *B. rapa CPA* gene family was found to have strong covariance with the *A. thaliana CPA* gene family, suggesting that highly homologous duplicative relationships may have arisen from the same gene duplication events.

### 2.7. Transcriptional Profiling of BrCPA Family Members in Response to Salt Stress

**Figure 6:**
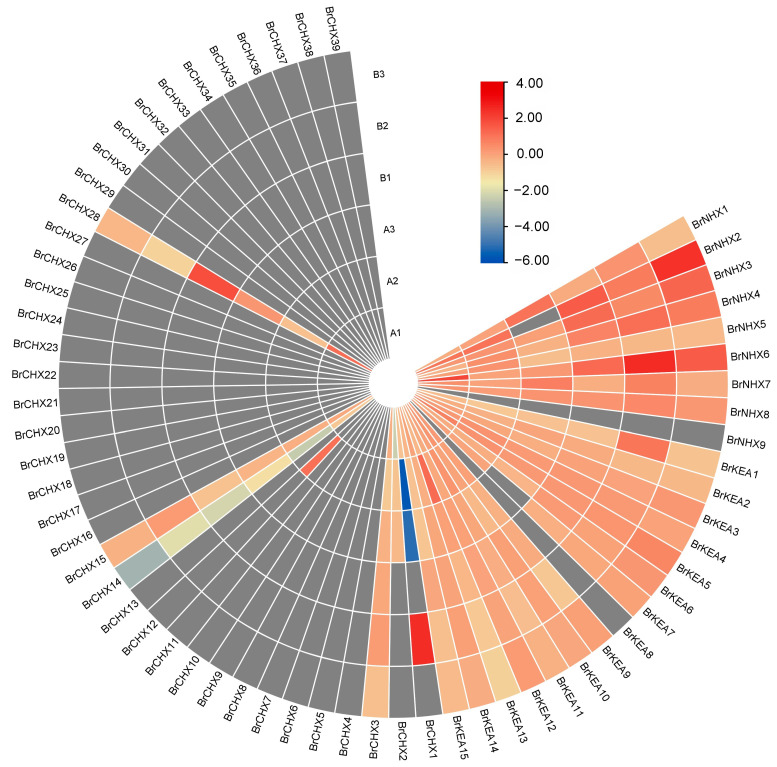
The expression of CPA family genes in *B. rapa* under salt stress in different varieties. Heatmaps are represented using log_2_ values for each gene. The color scale indicates the relative expression level from low (blue) to high (red).

Transcriptome data of two *B. rapa* varieties, ‘SCKY-6-27’ and ‘197-2018 Qin-10-45’, which differ in salt tolerance, were utilized to compare the transcriptional Profiling of *CPA* family members in *B. rapa* under different salt concentrations (Figure 6, Table S4). The results revealed that the expression of *CPA* family members in *B. rapa* showed significant differences between SCKY-6-27 and 197-2018 Qin-10-45 at different salt concentrations, but most of the family members had low or no expression. The salt-tolerant variety SCKY-6-27 had the highest expression of *BrCHX29* at 194 mmol/L salt concentration treatment and *BrCHX17* at 582 mmol/L salt concentration treatment. The highest expression of *BrCHX34* under 582 mmol/L salt treatment was observed in salt-sensitive variety 197-2018 Qin-10-45. The expression of *BrNHX4* in salt-tolerant variety SCKY-6-27 and salt-sensitive variety 197-2018 Qin-10-45 showed a trend of decreasing, then increasing, and then decreasing with an increase in salt concentration.

### 2.8. Expression Analysis of BrCPA Family Members Under Salt Stress

**Figure 7:**
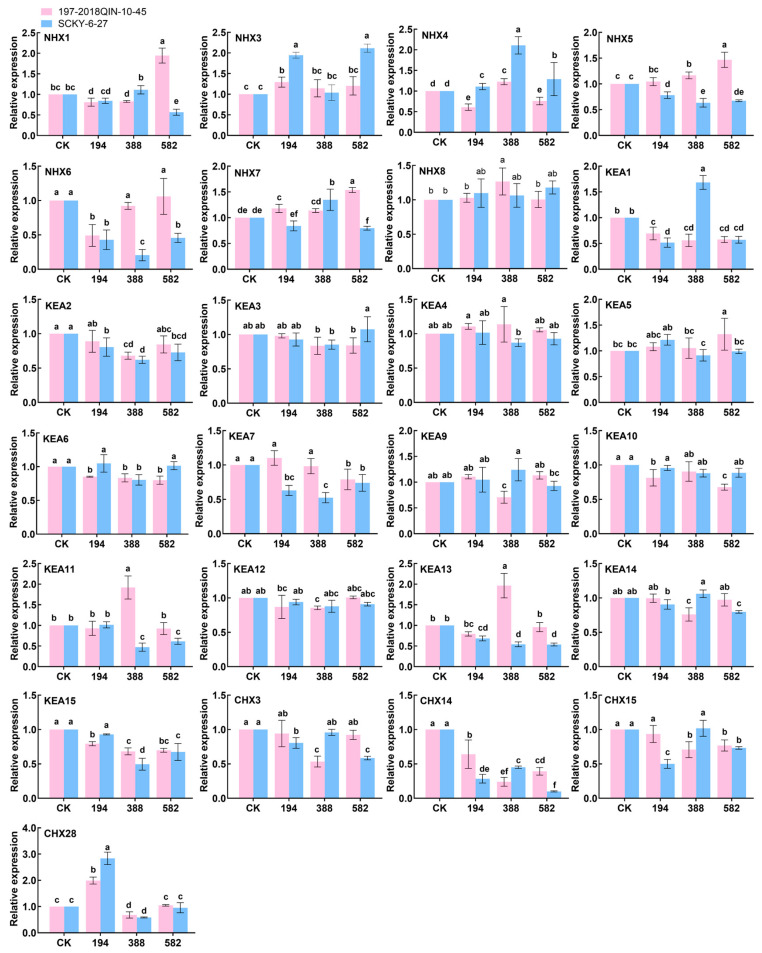
Expression profiles of *CPA* family genes in *B. rapa* under salt-stress conditions were investigated. The selected gene’s expression was assessed at salt concentrations of 0, 194 mmol/L, 388 mmol/L, and 582 mmol/L. Results, expressed as mean ± standard error from three biological replicates, are illustrated with error bars. Statistical significance (*p* ≤ 0.05) is indicated by lowercase letters.

Screening of genes from transcriptome data was conducted for the initial RT-qPCR detection (Figure 7, Table S5). To study the expression pattern of *CPA* genes under salt stress, the expression of *BrNHX4*, *BrCHX13*, and *BrCHX17* in “SCKY-6-27” was significantly higher than that in “197-2018QIN-10-45”, and the expression of *BrCHX7* in “197-2018QIN-10-45” was significantly higher than that in “197-2018QIN-10-45”. The expression of *BrCHX7* in “197-2018 Qin-10-45” was significantly higher than that of “SCKY-6-27” by 1.72, 28.2, 14, and 13.3 times. The expression of *BrCHX7* in “197-2018Qin-10-45” was 10.8-fold higher than that of “SCKY-6-27” in “197-2018Qin-10-45” under 582 mmol/L treatment.

## 3. Discussion

Salt stress causes ion imbalance and ionic toxicity in plants, as well as osmotic and oxidative stress, directly interfering with normal growth and developmental processes in plants [27]. Plants under salt stress mitigate the damage by regulating ion homeostasis and osmotic balance [28]. *CPA* family members have key roles in salt stress response, cell proliferation, ion homeostasis, and vesicle transport [29].

Na^+^ and K^+^ play crucial roles for all organisms. Previous studies have revealed that the Na^+^ efflux rate in salt-tolerant cultivars of *Brassica napus* is significantly higher than that in salt-sensitive cultivars under salt-stress conditions [30]. Our findings demonstrated that non-injury microtomography detection indicated a significant increase in Na^+^ and K^+^ efflux from the roots and leaf pulp of salt-sensitive varieties under salt stress compared to normal conditions. Furthermore, the efflux capacity of Na^+^ and K^+^ in the roots and leaf pulp of salt-sensitive varieties was markedly greater than that of salt-resistant varieties following salt stress. These findings suggest that salt-resistant varieties exhibit salt tolerance due to their enhanced capacity for Na^+^ and K^+^ retention. Dong et al. [31] investigated the Na^+^ and K^+^ flux rates in wheat under salt stress and found that salt stress increased the K^+^ efflux rate in seedlings of salt-sensitive wheat cultivars. Due to the salt exclusion mechanism, salt-tolerant cultivars exhibited stronger Na^+^ exclusion and Na^+^ compartmentalization, resulting in a lower Na^+^ efflux rate in salt-tolerant cultivars compared to salt-sensitive ones. The results show that although salt-tolerant *B. rapa* varieties did not show the ability of Na^+^ efflux than salt-sensitive varieties, they possessed strong Na^+^ rejection and Na^+^ compartmentalization because of salt rejection. The robust Na^+^ compartmentalization and K^+^ retention capabilities of salt-tolerant *B. rapa* variants are the primary factors contributing to their significant salt tolerance.

The *CPA* gene family has been identified in a wide range of plant species, including maize [32] and allotetraploid rapeseed [29]. However, the distribution and biological functions of the *CPA* gene family in *B. rapa* have not been reported to date. In this study, a total of 63 *BrCPA* genes were identified. Phylogenetic analysis revealed that the CPA proteins in *B. rapa* are divided into three subfamilies, with the majority of these genes belonging to the *CHX* subfamily, which is largely consistent with findings in *A. thaliana* [15,16]. This indicates that the evolution and classification of CPA proteins are highly conserved across different species. The analysis of the physicochemical properties of BrCPA proteins revealed that the majority of the *CPA* family proteins exhibit strong hydrophobicity, while a minority show hydrophilicity. Additionally, there are significant differences in the molecular weights and isoelectric points of these proteins (Table S2). In studies on the physicochemical properties of proteins in crops such as *Amaranthus tricolor* [33] and radish [34], it was found that all proteins exhibit hydrophobicity. Studies have shown that the dynamic changes in the physicochemical properties of proteins may be closely related to their regulatory roles in the development of different plant tissues and organs, as well as their functions in hormone signal transduction [35]. It was found that CPA proteins are mainly localized to the plasma membrane, vesicle membrane, and organelle membrane of plant cells [18]. In this study, subcellular localization predictions revealed that most of the *BrCPA* gene family members were localized in cytoplasm, and a few family members were localized in the nucleus and chloroplasts.

Cis-acting elements, functioning as genetic switches for gene transcription, play a crucial regulatory role in biological processes, including responses to environmental stress and developmental stress [36,37]. Our results demonstrate that the cis-regulatory elements within the *BrCPA* promoter primarily include light-responsive, development-related, stress-responsive, and hormone-responsive elements. Light-responsive elements represent the largest category of cis-elements in *BrCPA*, which is consistent with the findings from studies on the *CPA* gene family in soybeans [17]. Additionally, the *BrCPA* promoter region also contains hormone-responsive and stress-responsive elements.

During plant evolution, gene duplication facilitates functional innovation or diversification through neofunctionalization or subfunctionalization mechanisms, providing a genetic basis for plants to adapt to environmental changes. This process enhances their survival advantages under environmental stress and improves adaptability through functional specialization [38]. According to the intra-species collinearity analysis of *B. rapa*, the members of the *CPA* family in *B. rapa* are associated with segmental or tandem duplications, indicating that gene duplication plays a significant role in the expansion of the *CPA* gene family in the *B. rapa* genome. Tandemly repeated genes play a significant role in plant responses to environmental stress by regulating gene expression levels [39]. For example, the *NAC* transcription factor family in rice has expanded through whole-genome duplication events, and the duplicated gene members exhibit divergent expression patterns under drought stress, thereby enhancing the plant’s stress resistance [40].

Extensive studies have robustly demonstrated that members of the *CPA* gene family play a pivotal role in mediating responses to diverse abiotic stresses, including heavy metals, temperature fluctuations, and salt stress [41,42,43,44]. In these transcriptome data, it was found that some members of the *BrCPA* gene family were not expressed or had low expression levels under salt stress, with the majority belonging to the *CHX* subfamily. Among the three subfamilies of *BnaCPAs*, the *BnaKEA* and *BnaNHX* subfamilies exhibited a higher proportion of responses to nutrient stress, while the *BnaCHX* subfamily showed fewer members with differential expression between the control and stress conditions [29]. Therefore, we conclude that the *BrNHX* and *BrKEA* subfamilies are of significant importance to the abiotic stress resistance of the *BrCPA* family. Previous studies have found that overexpression of soybean *GmSOS1* in *A. thaliana* enhances salt tolerance by improving seed germination and plant growth under salt stress, accompanied by reduced Na^+^ accumulation, suggesting *GmSOS1* may limit Na^+^ uptake or enhance Na^+^ exclusion [45]. Real-time fluorescent quantitative PCR assay analysis clarified that the expression of *BrCHX14* was down-regulated in both ‘SCKY-6-27’ and ‘197-2018 Qin-10-45’, and the expression of *BrNHX6* was significantly higher than that of ‘SCKY-6-27’ in ‘197-2018 Qin-10-45. Through phylogenetic analysis, it was found that *AtCHX20* and *BrCHX14* are homologous genes, and *AtNHX5* and *AtNHX6* are homologous genes of *BrNHX6*, indicating that there is a certain similarity in the functions of these genes. The gene functions of *AtCHX20*, *AtNHX5*, and *AtNHX6* have been identified in *A. thaliana*. Research has shown that *AtNHX5* and *AtNHX6* play a role in promoting K⁺ transport in cells and are of great significance for K⁺ and pH homeostasis in *A. thaliana* [46]. *AtCHX20* plays a crucial role in osmotic regulation through K⁺ flux and the pH regulation of the active endomembrane system in guard cells [47]. These findings suggest that the *CPA* family genes may enhance the salt stress tolerance in *B. rapa*. This study also provides genetic resources for subsequent research on ion flux in *B. rapa*.

## 4. Materials and Methods

### 4.1. Experimental Materials and Salt Stress Treatments

In this experiment, two *B. rapa* germplasm with different salinity tolerances, i.e., the salt-tolerant material SCKY-6-27 (LC = 479 mmol/L) and the salt-sensitive material 197-2018 Qin-10-45 (LC = 315 mmol/L), were selected, which were provided by the *B. rapa* Research Group of Gansu Agricultural University. Seedlings, after 5–7 days of germination, were transferred to a new Petri dish to let their roots and cotyledons continue to grow, continued to cultivate for 7 days, and then transplanted to a hydroponic box containing Hoagland’s nutrient solution and the nutrient solution was added to the hydroponic box every 5 days to fill up the hydroponic box. *B. rapa* seedlings were placed in a greenhouse at a constant day/night temperature of 25 °C, light intensity of 6400 LX, and humidity of 30% (16 h of light, 8 h of dark). At the five-leaf stage of *B. rapa* seedlings, treatments were applied using mixed salt solutions of different concentrations. After three hours of salt and alkali stress treatment, the leaves of *B. rapa* were quickly collected, the samples were flash-frozen in liquid nitrogen and then transferred to an ultra-low temperature refrigerator at −80 °C for subsequent RNA extraction and reverse transcription experiments. The salt solution composition for salt stress was selected as NaCl, CaCl_2_, MgSO_4_, NaSO_4_, and NaHCO_3_, and the five salt NaCl: CaCl_2_: MgSO_4_: NaSO_4_: NaHCO_3_ = 40:2:4:16:1 molar ratio was mixed, and set to 0 (CK), 194 mmol/L, 388 mmol/L, and 582 mmol/L (Table 1).

### 4.2. Na^+^ and K^+^ Flow Rate Analysis

Changes in Na^+^/K^+^ efflux from the root elongation zone and chloroplasts of *B. rapa* under salt stress were measured using a non-invasive microtesting technique [48] to measure Na^+^ and K^+^ fluxes. The intact *B. rapa* root system and 1 cm × 0.2 cm-sized chloroplastic tissues under normal growth conditions were fixed at the bottom of Petri dishes, and then the roots and chloroplastic tissues were placed in test solution 1 (1.0 mM NaCl, 0.1 mM KCl, 0.2 mM MES, pH 5.8) and test solution 2 (1.0 mM NaCl, 1.0 mM KCl, 0.2 mM MES, pH 5.8) In the control test solution 1, *B. rapa* roots were left to stand for 0.5 h and leaf pulp tissues were left to stand for 4 h in the control test solution 2 in order to detect the sodium and potassium ion mobility rates in the elongation zone of the root system and the leaf pulp cells. As for detecting sodium and potassium ion flow rates under salt stress, the *B. rapa* materials were treated with 100 mM NaCl for 24 h, and then the intact *B. rapa* roots and 1 cm × 0.2 cm-sized chloroplastic tissues were fixed at the bottom of Petri dishes. To detect the sodium ion flow rate of the root system, the roots need to be placed in test solution 1 for 0.5 h and in test solution 3 (100 mM NaCl, 1.0 mM KCl, 0.2 mM MES, pH 5.8) for 0.5 h in order to detect the sodium and potassium ion flow rates in the roots, respectively. To detect the sodium ion flow rate of the leaf pulp cells, the leaf pulp tissues need to be placed in test solution 3 for 4 h and then transferred to test solution 1 to continue to be placed in the test solution for 0.5 h. The site to be measured was found under the microscope (root: the point on the root surface 900 μm from the apex of the root tip, which is the elongation zone; chloroplast: the surface of the chloroplast tissue), the Na^+^ or K^+^ flow rate microsensor was placed at the site to be measured, and detection was started. Instantaneous ion fluxes were recorded for 5 min for each sample, and eight biological replicates were tested for each group.

### 4.3. Genome-Wide Identification and Evolutionary Analysis of the CPA Gene Family in B. rapa

Genomic information of *B. rapa* (version 3.0) was downloaded from the Cruciferae database, and *A. thaliana* genomic information from the *A. thaliana* database TAIR. The Hidden Markov Model (HMM) of the CPA structural domain was downloaded from Pfam (http://pfam-legacy.xfam.org/, accessed on 10 November 2024) [33] and used Blast to screen for *CPA* family members in *B. rapa*. Further validation was carried out using Pfam and CDD [49] online analysis tools to eliminate the sequences to be selected that did not contain or had incomplete structural domains of the *CPA* gene family, resulting in 63 *CPA* members of *B. rapa*. The CPA protein sequences of *Brassica napus* and *A. thaliana* were compared and phylogenetically analyzed using ClustalW (https://www.genome.jp/tools-bin/clustalw, accessed on 10 November 2024) software. Phylogenetic trees were constructed using MEGA 7.0 [50] software with bootstrap set to 1000, and the Evolview (https://evolgenius.info/helpsite/qst1.html, accessed on 22 November 2024) [51], an online platform, was utilized to enhance the visualization of the phylogenetic trees.

### 4.4. Sequence Composition, Physicochemical Characteristics of Proteins, Chromosomal Distribution, Cis-Regulatory Elements, and Evolutionary Collinearity Analysis of CPA Gene Family Members

The conserved motifs within the CPA protein sequences were examined utilizing the MEME (http://meme-suite.org/tools/meme, accessed on 2 December 2024) tool [52]. The physicochemical characteristics of proteins encoded by the *CPA* gene family were predicted for all members utilizing the ExPASy (http://web.expasy.org/protparam/, accessed on 2 December2024) platform [53]. Subfine localization prediction was performed using the online software WOLF PSORT (https://wolfpsort.hgc.jp/, accessed on 15 December 2024) [54]. Intron–exon structure mapping of the *CPA* gene family members was performed using GSDS (http://gsds.cbi.pku.edu.cn, accessed on 22 December 2024). The location of *CPA* gene family members on the *B. rapa* chromosome was determined using TBtools V2.110 [55] software, and a feasibility analysis was performed. The 2000 bp sequence upstream of the gene start codon was selected by TBtools, and cis-acting element analysis was performed using PlantCare (http://bioinformatics.psb.ugent.be/webtools/plantcare/html/, accessed on 25 November 2024) [56]. The *CPA* gene family members in *Brassica napus* were analyzed using TBtools, and the covariance between Winter *B. rapa* and *A. thaliana* was analyzed using TBtools.

### 4.5. Expression Analysis of BrCPA Family Members

*BrCPA* gene family members were screened based on their gene IDs from transcriptome data measured in the laboratory (NCBI accession number: PRJNA1158557). Total RNA from treated leaves of ‘SCKY-6-27’ and ‘197-2018 Qin 10-45’ was extracted using an RNA extraction kit (TIANGEN Biotech Co., Ltd., DP419, Beijing, China). Total RNA from treated leaves of ‘SCKY-6-27’ and ‘197-2018 Qin 10-45’ was extracted according to the manufacturer’s instructions, and the integrity and concentration of RNA were detected using 1% agarose gel electrophoresis and ultra-microspectrophotometer Nanodrop ND-2000 (Nanodrop Technologies, Wilmington, NC, USA). Subsequently, cDNA was obtained by reverse transcription using a reverse transcription kit (TIANGEN Biotech Co., Ltd., KR118-02, Beijing, China). cDNA was subjected to real-time fluorescence quantitative PCR under salt stress using the TIANGEN Fast Real Rapid Fluorescence PCR Premix Reagent Kit (TIANGEN Biotech Co., Ltd., FP217-02, Beijing, China) for RT-qPCR analysis. The relative expression of the gene was calculated using the 2^−ΔΔCt^ method. The primer sequences used were shown in Table S1, and each sample was repeated three times.

### 4.6. Data Analysis

Data were organized using Microsoft Excel 2019 software, analyzed by ANOVA using SPSS 25.0 software, multiple comparisons using the Duncan method, and graphing was completed using Origin 2021 and GraphPad Prism 10 software.

## 5. Conclusions

In this study, a total of 63 *BrCPA* genes were identified in the genome of *B. rapa*, which were classified into three subfamilies, and the characteristics exhibited by the members of the *BrCPA* gene family were systematically analyzed. The physicochemical properties of the *BrCPA* families were found to vary significantly, while the conserved sequences of gene results and proteins were highly conserved. Chromosomal distribution showed that *BrCPA* gene family members were unevenly distributed on all chromosomes of *B. rapa*. Comprehensive analysis of gene structure and conserved structural domains revealed that the exons of *CHX* family members were shorter, while those of *NHX* and *KEA* family members were longer, and all the members did not contain UTR regions, and it was found by subcellular localization that most of the members of the *BrCPA* gene family were located in the cytoplasm, and that most of the *BrCPA* gene members from the same subfamily showed the same motifs and cis-regulatory The expression levels of *BrCPA* genes were different under different salt concentrations, and most members of the *CHX* subfamily had low or no expression, suggesting that their members may not be involved in regulation under salt stress, and the genes with higher expression were verified and analyzed by RT-qPCR. Under salt stress, salt-sensitive varieties exhibit higher Na^+^ and K^+^ efflux rates in roots and mesophyll tissues, which are significantly greater than those observed in salt-tolerant varieties. Non-invasive microtesting techniques can be applied as a new technique for screening salt-tolerant varieties of *B. rapa* and breeding for salt tolerance. This research lays the theoretical groundwork for future investigations into the function and action mechanism of the CPA gene in *B. rapa* plant growth and development.

## Figures and Tables

**Table 1:** Names corresponding to the transcriptome.

Breed Name	Treatment Concentration (mmol/L)	Analysis Name
197-2018 QIN 10-45	0	A4
197-2018 QIN 10-45	194	A3
197-2018 QIN 10-45	388	A2
197-2018 QIN 10-45	582	A1
SCKY-6-27	0	B4
SCKY-6-27	194	B3
SCKY-6-27	388	B2
SCKY-6-27	582	B1

## Data Availability

Data from this study can be found in the article and Supplementary Materials.

## Supplementary Materials

The following supporting information can be downloaded at: https://www.mdpi.com/article/10.3390/ijms26073099/s1.
