# Supplementary material for: Genome-Wide Identification of the Cation/Proton Antiporter (CPA) Gene Family and Expression Pattern Analysis Under Salt Stress in Winter Rapeseed (Brassica rapa L.)
Source: Int J Mol Sci. 2025 Mar 27;26(7):3099. doi: 10.3390/ijms26073099 (PMC11988609; doi:10.3390/ijms26073099)
Supplement: Supplementary file 1 [file ijms-26-03099-s001.zip › Table S1.pdf]

Table S1 Primer sequence information of saline-alkali stress response genes sequences

Gene ID	Primer sequence 5'-3'	Primer sequence 3'-5'
BrNHX1	TCGGAGAAGGTGTTGTGAATGATG	GTTAAGGTGGGTGAGGTCAAAGC
BrNHX3	GTGTTCTTCTGTGGTATTGTGATGTC	AAGGTATGCTTGGTAGTTACTCTTGAG
BrNHX4	ACCGTCGTTCTCCTCGTCATC	GTAGGTCAGGGTTAATATCGTTCCAG
BrNHX5	TTCGCCGTCACAAGTTCTATTACC	GTCCTTATGCTAGTCTCCGTGTTC
BrNHX6	TCGTTGGTGGTTTAGCGAAC	AGAGAGCCAAACATGAGACAC
BrNHX7	TGTGTTCTTCTGTGGGATTGTGATG	GGCGTGCTTGGTAGTGATTCTTG
BrNHX8	AGGAGGCGACAGGTTCTTCTTC	ATACCGAGCACCAGAGACATCC
BrNHX9	TCAAGCAGTTATACGCACCAC	GATGGTGTAAACAGGCTGTTC
BrKEA1	TCTCCTCTTACTCTCATTCTTCCTCTG	AGTTGGCGACGGCTTCCTC
BrKEA2	TCCGTAATTTGTTTGCCGCTCTC	GACTAGAATCACAGATGCCAGAAGG
BrKEA3	CCAAGACTGATAGATAGGAAGGACAAC	CAGAAACAATGACGACGACCAAATC
BrKEA4	ATACGCACATACGCACGTTTC	GAGTATCTTCTACGCCATTCTC
BrKEA5	ATACGCACATACGCACGTTTC	GAGTATCTTCTACGCCATTCTC
BrKEA6	TATCGGGACTGAGTCAGTTG	CCTTTGGTCGTGCGTATTTG
BrKEA7	ATACGCACATACGCACTAGC	AGCAAAAAGCAAACCCACAGC
BrKEA9	CACACTCTTGGCTGGTCTGATTG	AACCGATTCTTACGCTTTCTTGGAG
BrKEA10	TGACAAGCCTTTCTTCTCAGACTAA	ACTACACCAAGCGACAAGCAAG
BrKEA11	GGCACAACAACACTGAGCAGAG	TCCAACATTCTTCATTCCTCCTACTTC
BrKEA12	GACAGCGGCAGTAGTTGGATTG	AACAGCGGTGGAGGACAGTG
BrKEA13	TTGCGAGTCTGGTTGATTCTGTC	TAATGTCAGTCCTGCTCCGAGTAG
BrKEA14	TGACAGCAGCAGTAGTTGGATTG	AGGACAACAGCAGTGGAGGAC
BrKEA15	TCAACAACAGATGTAGCTGAGG	AGTATACAATGACAACCACCTAGG
BrCHX3	TCGTTGGTTAGCAGTACGGAT	TTGATCCAGTCAACCTGTCTC
BrCHX14	GCATGGCAAGGCGACAATCC	CGACTGACGGCGATGATTAACG
BrCHX15	TGGAAGGAGGAGGAAGAAGAAGAAG	GAGGCGATGGAGAAGCAGAGG
BrCHX28	ATACGCACGAATCATGGTCAATGG	TGCGTTATCTCAACTTTTCCCC
Brapa09g000438（PP2A）	AGGGCTATCACCTTCTC	ACACATTGGTCCTTCGT
